# Use of abrocitinib in severe atopic dermatitis

**DOI:** 10.1590/1806-9282.2025D722

**Published:** 2026-04-20

**Authors:** Maria Fernanda Mollaco Navarro da Cruz, Giulia Jardim Criado, Thais Alice Ortrolani, Marcia da Silva Martins, Sandra Lopes Mattos e Dinato, Antonio Silvinato, Wanderley Marques Bernardo

**Affiliations:** 1 Centro Universitário Lusíada, Faculty of Medical Sciences of Santos, Department of Evidence-Based Medicine – Santos (SP), Brazil.; 2 Centro Universitário Lusíada, Faculty of Medical Sciences of Santos – Santos (SP), Brazil.; 3 Unimed Santos Clinical Governance Center – Santos (SP), Brazil.; 4 Centro Universitário Lusíada, Department of Dermatology – Santos (SP) Brazil.; 5 Brazilian Medical Association, Department Evidence-Based Medicine – São Paulo (SP), Brazil.; 6 Universidade de São Paulo, Faculty of Medicine – São Paulo (SP), Brazil.

## INTRODUCTION

Atopic dermatitis is a chronic inflammatory skin disease characterized by eczematous lesions associated with intense itching. It is a common childhood dermatosis, with 85% of cases starting before the age of 5 and over 70% disappearing before adolescence. These patients often have other comorbidities, which make up the atopy triad: allergic rhinitis and asthma, with atopic dermatitis (AD) usually being the first clinical manifestation^1^.

It is considered to have a multifactorial etiology, resulting from the interaction between genetic and environmental factors. This combination leads to an immune imbalance characterized by the predominance of a Th2-type response in the acute phase, with increased expression of cytokines such as interleukin (IL)-4, IL-5, and IL-13, which contribute to maintaining the inflammatory process and pruritus^1^.

AD can manifest itself in different regions of the body and shows wide clinical variability between individuals. Among the main signs and symptoms observed are skin xerosis, pruritus, exudation with crust formation, skin thickening with lichenification, and periorbital hyperpigmentation^2^.

The disease can significantly compromise the quality of life of patients and their families, especially during episodes of acute clinical exacerbation. Itching, its main debilitating symptom, is often associated with sleep disturbances, impaired school and work activities, emotional impairment, and reduced social interaction, resulting in significant physical, psychological, and social impacts.

For this reason, assessing the severity of AD is essential both for selecting the appropriate therapy and for monitoring the response to treatment, given that this can vary over time.

There are assessment instruments used both in clinical practice and in research, which measure various aspects related to the disease. The most widely used and validated scores are the Scoring Atopic Dermatitis and the Eczema Area and Severity Index. In addition to these, AD-related quality of life can be assessed using the Dermatology Life Quality Index, especially in adults. It is also recommended that the severity be assessed by the patient themselves, with the Patient-Oriented Eczema Measure being the most widely used instrument for this purpose^3^.

The aim of treating AD is to reduce symptoms and control the disease in the long term. Those with moderate to severe AD, with a partial response to topical and systemic treatment using methotrexate and cyclosporine, are referred for treatment with immunobiologicals: monoclonal antibodies (such as dupilumab) and Janus-kinase inhibitors (such as abrocitinib, baricitinib, and upadacitinib)^4^.

Dupilumab was considered a watershed in its treatment and is currently the immunobiological of choice. It is a monoclonal antibody approved for use by the American Food and Drug Administration, the European Medicines Agency, and, in Brazil, by Anvisa (National Health Surveillance Agency) in 2018 for the treatment of adults, and in 2023, it will be released for patients over the age of 6 months^4^.

Although dupilumab is currently the main immunobiological therapy approved for the treatment of moderate to severe AD, some of its characteristics should be considered, such as the need for subcutaneous administration at regular intervals and an onset of action that, although effective, tends to occur gradually over weeks^5^.

Abrocitinib, on the other hand, is a selective Janus-kinase 1 (JAK1) inhibitor, administered orally that blocks the signaling of various cytokines involved in the pathophysiology of AD, such as interleukin-4, interleukin-13, and interleukin-31. According to international guidelines and regulatory documents, abrocitinib has the potential to provide a faster reduction in pruritus and clinical improvement in skin lesions, in addition to the convenience of oral administration^6^.

Thus, considering the differences in the administration profile, clinical response time, and mechanism of action, this study was carried out with the aim of assessing whether abrocitinib is an effective and safe alternative for the treatment of moderate to severe rash, just like dupilumab, whose efficacy and safety have already been widely established.

## OBJECTIVE

The aim of this study was to evaluate the efficacy and safety of abrocitinib in the treatment of moderate and severe AD in patients aged 12 years and over.

## METHODS

This systematic review and meta-analysis followed the precepts of Preferred Reporting Items for Systematic reviews and Meta-Analyses (PRISMA)^7^ (Supplementary Figure 1) and is registered with PROSPERO (international prospective register of systematic reviews)^8^ under registration CRD420251071443.

A systematic literature review was carried out using the Medline, Embase, ClinicalTrials, and Google Scholar databases. The following search strategy was used: (Dermatitis, Atopic OR Atopic Dermatitides OR Atopic Dermatitis OR Atopic Neurodermatitides OR Atopic Neurodermatitis OR Disseminated Neurodermatitides OR Disseminated Neurodermatitis OR Atopic Eczema) AND (abrocitinib) AND Random*. The scientific evidence selected was that which met the following eligibility criteria:

- Population: Patients ≥12 years;
- Intervention: Abrocitinib;
- Comparison: Placebo and dupilumab;
- Outcome: Efficacy and safety;
- Study design: Phase III randomized clinical trial (RCT); without period or language limits; with full text or abstract with available data.

The data extracted from the selected articles were age, disease severity, number of patients using abrocitinib, dupilumab, and placebo, dosage, time of application of abrocitinib and dupilumab, follow-up, Investigator's Global Assessment (IGA) response at 12 weeks and serious adverse effects related to the drug.

The selected evidence was critically evaluated by investigating the risk of bias using RoB 2.0^9^, which included: randomization, blinded allocation, double blinding and blinding of the evaluator, losses, prognostic characteristics, measurement of outcomes, intention-to-treat analysis, sample calculation, and early discontinuation. Risk was estimated as very high, high, or low.

The quality of the evidence analysis was expressed as very low, low, moderate and high. The items considered (GRADEpro^10^ software) were classified as very high, high and low, taking into account the following items: risk of bias, inconsistency, precision, indirect evidence and publication bias.

The extracted data were meta-analyzed using RevMan 5.4 software^11^ The results (efficacy and safety) were expressed as risk difference, with a 95% confidence level and number needed to treat (NNT) or to produce harm. Heterogeneity in I^2^ ranges from 0 to 100%, with values above 50% considered high (inconsistency). Random (I^2^>50%) and fixed (I^2^≤50%) models were used for analysis.

## RESULTS

The search conducted up to August 2025 retrieved 81 publications (with limits for RCTs), from which 32 studies were initially selected. Meeting the eligibility criteria, three studies^12–14^ were included to support this evaluation (Supplementary Figure 1).

The three randomized clinical trials selected included two different age groups: 12–17 years (n: 285) and ≥18 years (n: 1,565). The total number of patients studied was 1,850, with 227 receiving placebo, 1,015 abrocitinib, and 608 dupilumab.

Of the patients treated with abrocitinib, 683 received the 200 mg dose and 332 the 100 mg dose. As for dupilumab, all 608 patients received the standard dose of 600 mg subcutaneously initially, followed by 300 mg every 2 weeks, according to the approved protocol.

Follow-up times varied between the studies, with 12-, 16-, and 26-week follow-ups.

Outcomes assessed: IGA (0–1 and/or improvement ≥2 points) at 12 weeks and drug-related adverse events (TEAEs) comparing abrocitinib to placebo and dupilumab.

The data from the two studies carried out in adults were combined in a meta-analysis evaluating drug-related adverse events, since the comparison between abrocitinib and dupilumab or placebo showed homogeneous results in this age group.

With regard to IGA at 12 weeks, the studies combined in a meta-analysis were Eichenfield et al.^12^ and Bieber et al.^13^, involving both adolescents and adults in their evaluation of abrocitinib and placebo.

The quality of evidence is moderate for outcomes comparing abrocitinib with placebo (Supplementary Table 1—APPENDIX), and high for drug-related adverse events when comparing abrocitinib with dupilumab (Supplementary Table 2). The risk of bias for one study has some concerns, and for the other two studies, it is considered low (Supplementary Table 3).

In patients over the age of 12 with moderate to severe AD unresponsive to conventional treatment, the use of abrocitinib as an alternative therapy to dupilumab produced the following effects:

### Investigator's Global Assessment (0–1 and/or improvement ≥2 points)

In this analysis (Supplementary Figure 2), 313 patients receiving abrocitinib 200 mg and 225 in the placebo group were studied. Increase in improvement (measured by IGA) of 29%, ranging from 17 to 41% (95%CI), with four patients having to be treated for ≥12 years to obtain 1 improvement ≥2 points or with a score between 0 and 1 (NNT: 4). The quality of evidence is moderate (Supplementary Table 1).

### Drug-related adverse events (TEAEs): abrocitinib×placebo

In this analysis (Supplementary Figure 3), 320 patients undergoing abrocitinib and 227 in the placebo group were studied. There was no difference in the risk of TEAEs with abrocitinib treatment compared to placebo. The quality of the evidence is moderate (Supplementary Table 1).

### Drug-related adverse events (TEAEs): abrocitinib×dupilumab

In this analysis (Supplementary Figure 4), 588 patients undergoing abrocitinib and 607 in the placebo group were studied. There was no difference in the risk of TEAEs with treatment with abrocitinib compared to dupilumab. The quality of the evidence is high (Supplementary Table 2).

## DISCUSSION

This systematic review and meta-analysis showed a significant difference in favor of abrocitinib when compared to placebo in relation to IGA improvement, with a substantial increase in the proportion of patients achieving IGA 0 or 1 with improvement ≥2 points, with an NNT of 4. However, it was not possible to carry out this same comparative assessment between abrocitinib and dupilumab, since there is not enough homogeneous data to establish a clear superiority of the JAK inhibitor over the already well-established therapy with dupilumab.

As for TEAEs, the studies did not show a significant difference when comparing abrocitinib to either dupilumab or placebo, indicating that the drug studied has an acceptable safety profile and no relevant increase in risk in the context of the trials evaluated. The most common adverse events associated with abrocitinib were nausea, headache, acne, upper respiratory tract infections, and transient thrombocytopenia, while for dupilumab, conjunctivitis, injection site reactions, eosinophilia, and respiratory infections stood out, most of which were characterized as mild, self-limiting, and easily managed clinical adverse effects.

In addition to the efficacy and safety data presented in the meta-analysis, the study by Cork et al.^15^ makes a relevant contribution by analyzing the long-term safety of abrocitinib in different age groups. The results indicate that the drug has a manageable safety profile, with a lower incidence of adverse events in adolescents and numerically higher rates in patients aged 65 and over, especially at the 200 mg dose. These findings reinforce the importance of careful dose selection, especially in elderly patients, in order to reduce risks such as serious infections, thrombocytopenia, lymphopenia, and cardiovascular events. The analysis also suggests that, despite these risks in more vulnerable populations, abrocitinib remains a safe option, as long as the appropriate dose and clinical monitoring recommendations are respected, in line with the observations of the meta-analysis on the acceptable safety profile of the medication.

Thus, although current data supports the use of abrocitinib in different age groups, it is essential to note that its regulatory approval is restricted to patients aged 12 and over. There are still no conclusive studies attesting to its safety and efficacy in children under this age group, which reinforces the importance of its use strictly following the current indications until new scientific evidence expands this recommendation.

When comparing abrocitinib to dupilumab in terms of a possible superiority in terms of convenience, as it is an orally administered drug, the studies analyzed showed no evidence that this difference had a negative impact on adherence to or acceptance of treatment with dupilumab. On the contrary, the rate of discontinuation of the medication was similar between the two groups, indicating that subcutaneous administration does not, in practice, constitute a significant unfavorable condition in the management of moderate to severe AD.

Clinical response time was another aspect assessed in the studies. In comparisons between the drugs, abrocitinib was found to reduce pruritus more quickly than dupilumab. In the study by Bieber et al.^13^, the significant improvement in pruritus, assessed by the PP-NRS4 (Peak Pruritus Numerical Rating Scale), occurred early in the patients who used abrocitinib. Among those who received dupilumab, this response was later. Consistently, Reich et al.^14^ also identified a temporal difference favorable to abrocitinib in obtaining symptomatic relief related to pruritus in the early phases of treatment.

However, despite these observations, the comparison between the two therapies in terms of the speed of improvement in pruritus should be interpreted with caution, since the methodological differences between the studies, including the heterogeneity of the populations evaluated and the variation in follow-up times, make it difficult to draw definitive and widely applicable conclusions.

The limitations of this analysis include the fact that it was based on only three RCTs, which affects the generalizability of the results. In addition, the lack of direct and homogeneous comparisons between abrocitinib and dupilumab prevented the preparation of a meta-analysis that could more accurately assess the relative efficacy of these medications.

Another relevant aspect to consider is that all the RCTs included in this analysis were sponsored by Pfizer, the pharmaceutical company responsible for producing abrocitinib, and, to date, there are no other independent studies available in the literature that do not have this institutional link. This represents a potential source of bias and reinforces the need for new, independently conducted studies to validate and expand the evidence currently available on the use of abrocitinib in the treatment of moderate to severe AD.

## CONCLUSION

The findings analyzed indicate that abrocitinib has significant efficacy in improving the clinical scores of atopic dermatitis and maintains a safety profile comparable to that observed with placebo and dupilumab in the studies evaluated. In addition, the data suggest an earlier clinical response in relation to pruritus, a characteristic that could represent a relevant clinical differential in practice. However, the lack of direct, homogeneous, and independent comparisons between the two therapies limits the possibility of establishing definitive conclusions about differences in efficacy or safety. Thus, additional studies, especially head-to-head clinical trials and long-term analyses, are needed to confirm these preliminary results, broaden the understanding of the benefits and risks of abrocitinib, and guide clinical decision-making more consistently.

## Data Availability

The datasets generated and/or analyzed during the current study are available from the corresponding author upon reasonable request.
