# Supplementary material for: Use of abrocitinib in severe atopic dermatitis
Source: Rev Assoc Med Bras (1992). 2026 Apr 20;72(2):e2025D722. doi: 10.1590/1806-9282.2025D722 (PMC13108834; doi:10.1590/1806-9282.2025D722)

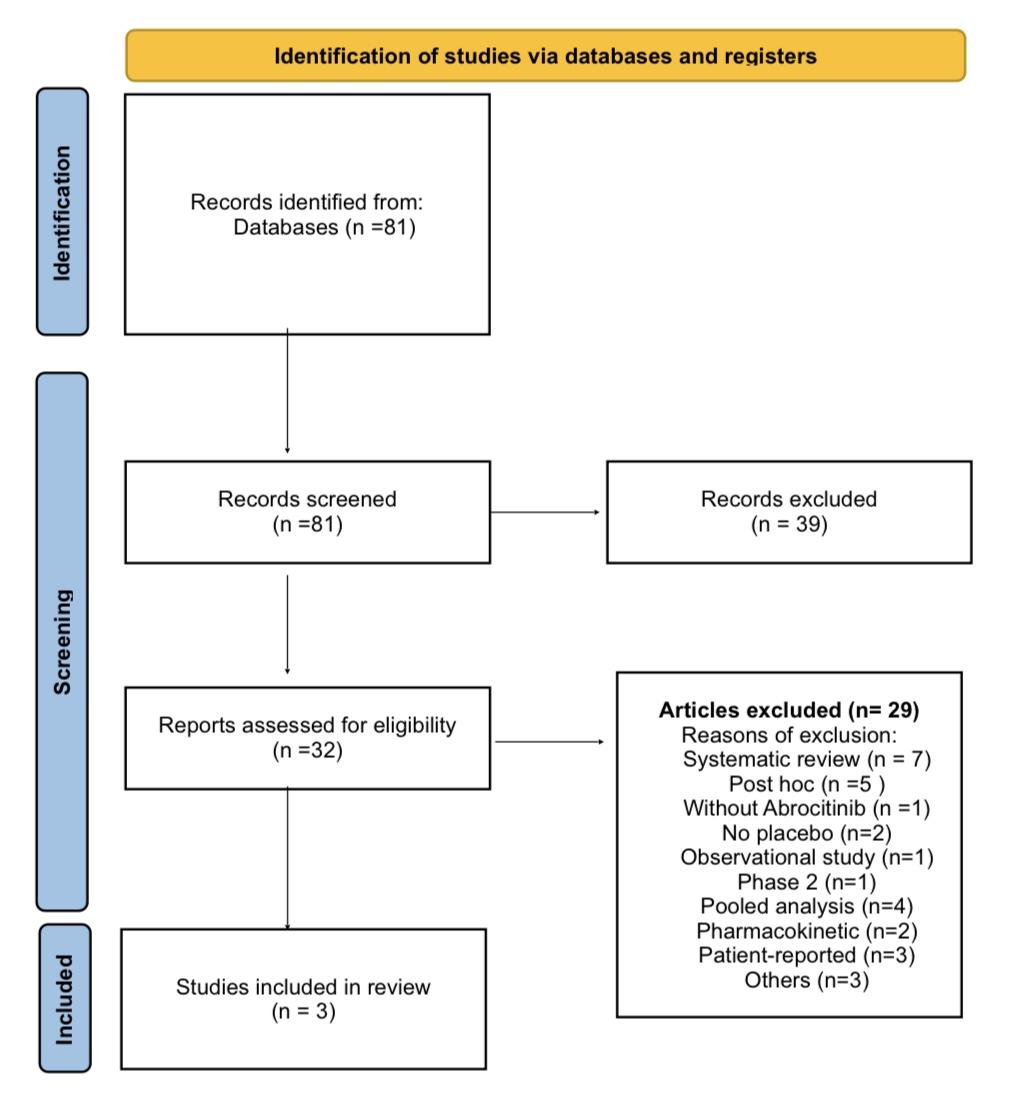
**APPENDIX**

**Supplementary Figure 1.** PRISMA follow diagram.


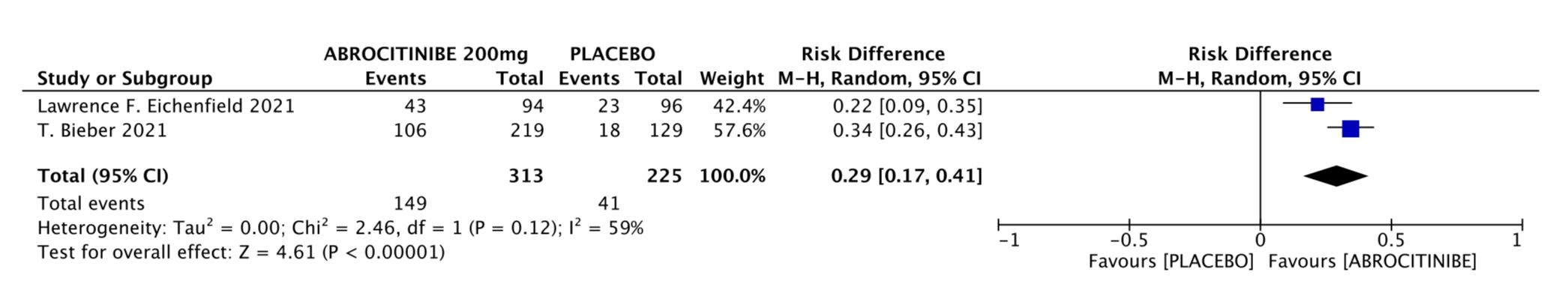


**Supplementary Figure 2.** Forrest plot—Investigator’s Global Assessment 12 weeks.


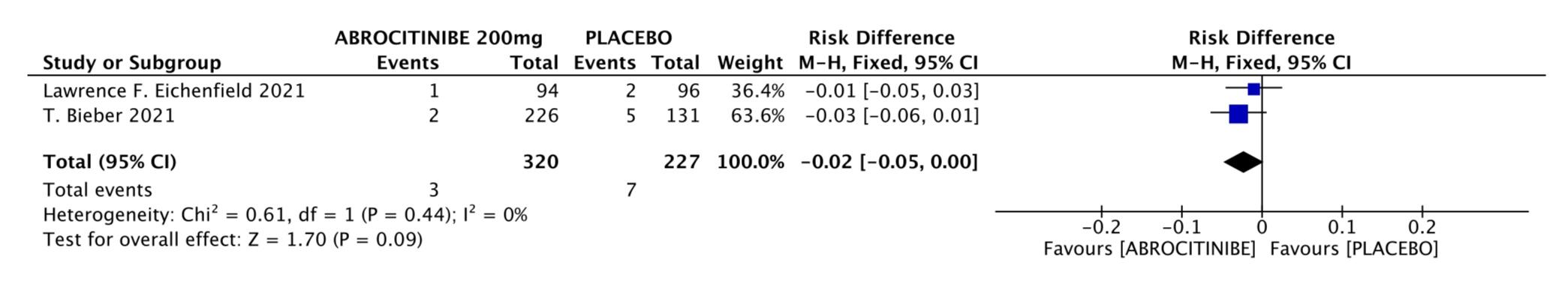


**Supplementary Figure 3.** Forrest plot—treatment-emergent **
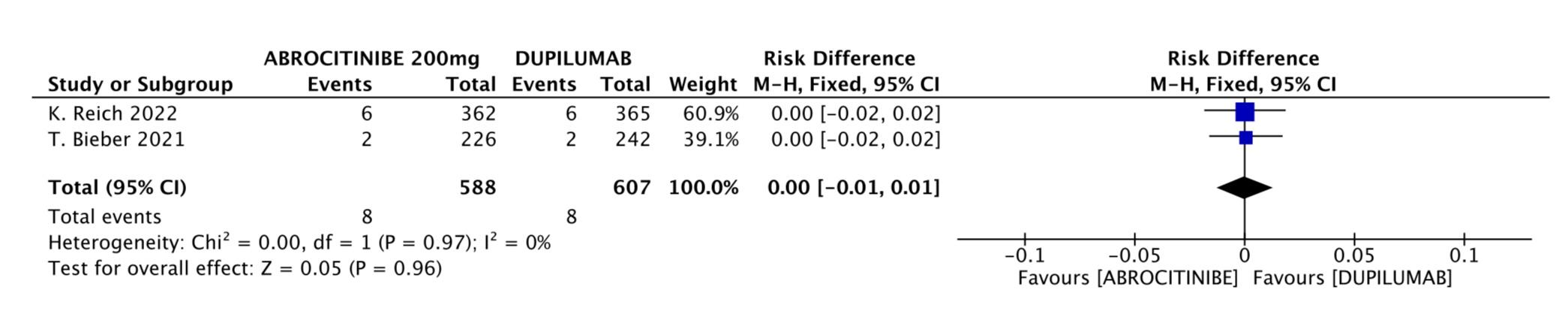
**adverse events (abrocitinib×placebo).

**Supplementary Figure 4.** Forrest plot—treatment-emergent adverse events (abrocitinib×dupilumab).

**Supplementary Table 1.** Quality of evidence (GRADEpro)—abrocitinib versus placebo.

**
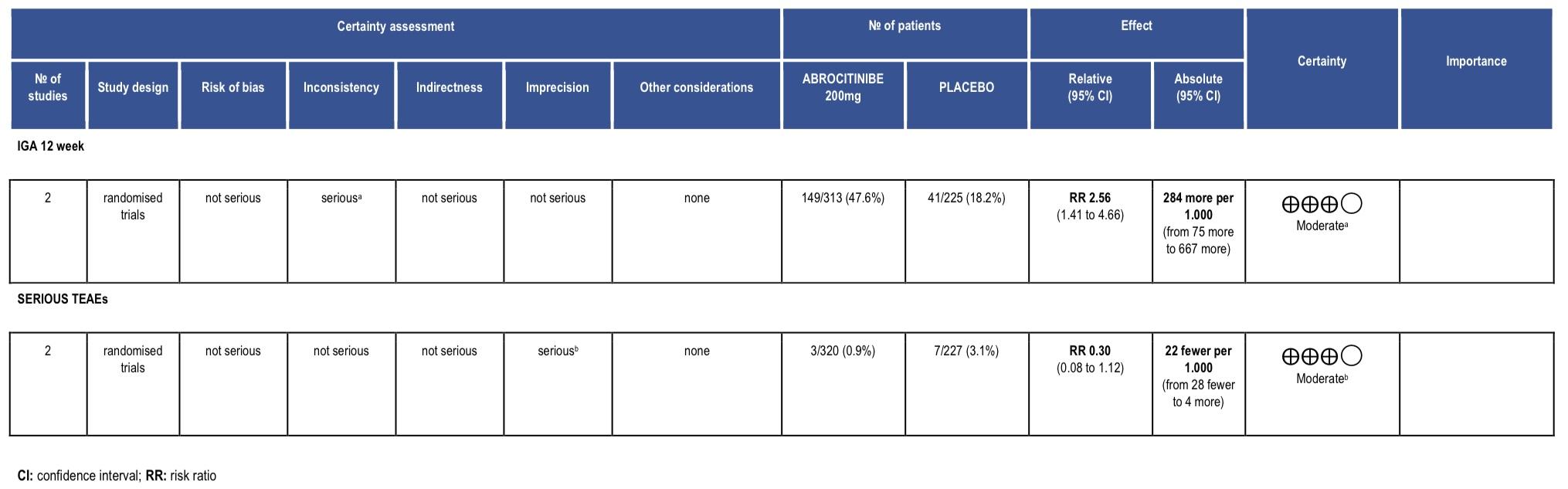
**

**Supplementary Table 2.** Quality of evidence (GRADEpro)—Abrocitinib versus dupilumab.


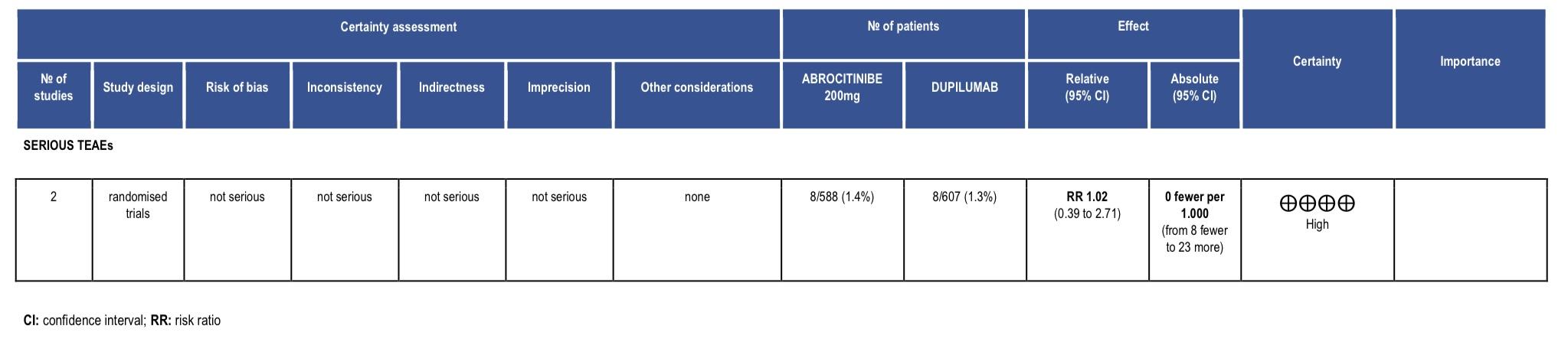


**Supplementary Table 3.** Risk of bias (RoB 2.0).


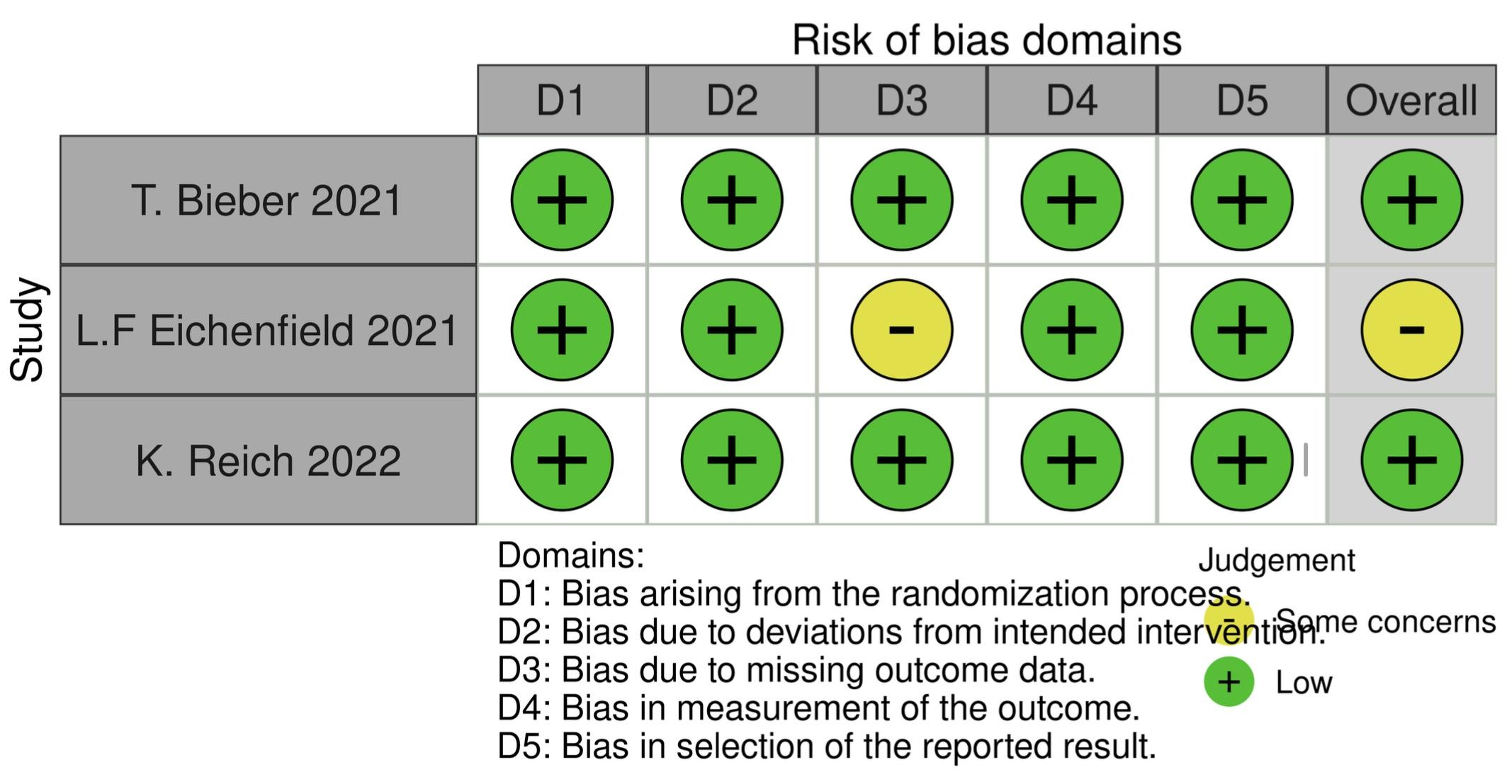

Supplement: Supplementary Material [file 1806-9282-ramb-72-02-e2025D722-suppl1.docx]
